# Human and *Drosophila* Cryptochromes Are Light Activated by Flavin Photoreduction in Living Cells 

**DOI:** 10.1371/journal.pbio.0060160

**Published:** 2008-07-01

**Authors:** Nathalie Hoang, Erik Schleicher, Sylwia Kacprzak, Jean-Pierre Bouly, Marie Picot, William Wu, Albrecht Berndt, Eva Wolf, Robert Bittl, Margaret Ahmad

**Affiliations:** 1 Université Paris VI, UMR-CNRS 7632, Paris, France; 2 Freie Universität Berlin, Fachbereich Physik, Berlin, Germany; 3 Institut de Neurobiologie Alfred Fessard, CNRS UPR 2216 (NGI), Gif-sur-Yvette, France; 4 Department of Structural Biology, Max Planck Institute of Molecular Physiology, Dortmund, Germany; 5 Penn State University, Media, Pennsylvania, United States of America; University of Geneva, Switzerland

## Abstract

Cryptochromes are a class of flavoprotein blue-light signaling receptors found in plants, animals, and humans that control plant development and the entrainment of circadian rhythms. In plant cryptochromes, light activation is proposed to result from photoreduction of a protein-bound flavin chromophore through intramolecular electron transfer. However, although similar in structure to plant cryptochromes, the light-response mechanism of animal cryptochromes remains entirely unknown. To complicate matters further, there is currently a debate on whether mammalian cryptochromes respond to light at all or are instead activated by non–light-dependent mechanisms. To resolve these questions, we have expressed both human and *Drosophila* cryptochrome proteins to high levels in living Sf21 insect cells using a baculovirus-derived expression system. Intact cells are irradiated with blue light, and the resulting cryptochrome photoconversion is monitored by fluorescence and electron paramagnetic resonance spectroscopic techniques. We demonstrate that light induces a change in the redox state of flavin bound to the receptor in both human and *Drosophila* cryptochromes. Photoreduction from oxidized flavin and subsequent accumulation of a semiquinone intermediate signaling state occurs by a conserved mechanism that has been previously identified for plant cryptochromes. These results provide the first evidence of how animal-type cryptochromes are activated by light in living cells. Furthermore, human cryptochrome is also shown to undergo this light response. Therefore, human cryptochromes in exposed peripheral and/or visual tissues may have novel light-sensing roles that remain to be elucidated.

## Author Summary

Vision in animals is generally associated with light-sensitive rhodopsin pigments located in the eyes. However, animals ranging from flies to humans also possess ancient visual receptors known as cryptochromes in multiple cell types. In this work, we study the mechanism of light sensing in two representative animal cryptochromes: a light-sensitive *Drosophila* cryptochrome (Dmcry) and a presumed light-insensitive mammalian cryptochrome from humans (Hscry1). We expressed recombinant cryptochromes to high levels in living cells, irradiated the cells with blue light, and analyzed the proteins' response to irradiation with electron paramagnetic resonance and fluorescence spectroscopic techniques. Photoreduction of protein-bound oxidized FAD cofactor to its radical form emerged as the primary cryptochrome photoreaction in living cells, and was correlated with a light-sensitive biological response in whole organisms. These results indicate that both Dmcry and Hscry1 are capable of undergoing similar light-driven reactions and suggest the possibility of an as-yet unknown photo-perception role for human cryptochromes in tissues exposed to light.

## Abstract 3

Cryptochromes are blue-light-absorbing receptors found in plants, animals, and humans. In mammals, they are not thought to respond to light, but this study demonstrates contrary evidence that indeed, human cryptochromes undergo a photochemical transformation in response to light.

## Introduction

Cryptochromes are blue-light–absorbing photoreceptors found throughout the biological kingdom, involved in diverse and important signaling roles [1–3]. Cryptochromes were first identified in plants from a mutant of Arabidopsis thaliana (A. thaliana), *hy4*, which failed to show normal plant growth and developmental responses to blue light [4]. The N-terminal region of the *HY4* encoding protein, renamed cryptochrome or A. thaliana cry1 (Atcry1) was found to be highly homologous to a previously characterized class of enzymes, DNA photolyases, which utilizes blue light as a source of energy for the repair of UV-light–generated DNA lesions [2,5]. However, cryptochrome did not repair DNA but instead, participated in numerous blue-light–dependent plant growth responses, including early seedling development, leaf and stem expansion, initiation of flowering, and gene regulation [6,7]. The defining characteristic of a cryptochrome-type photoreceptor is therefore a light receptor molecule that is structurally highly similar to DNA photolyases, but has lost DNA repair activity and acquired a novel role in signaling.

**Figure 1:**
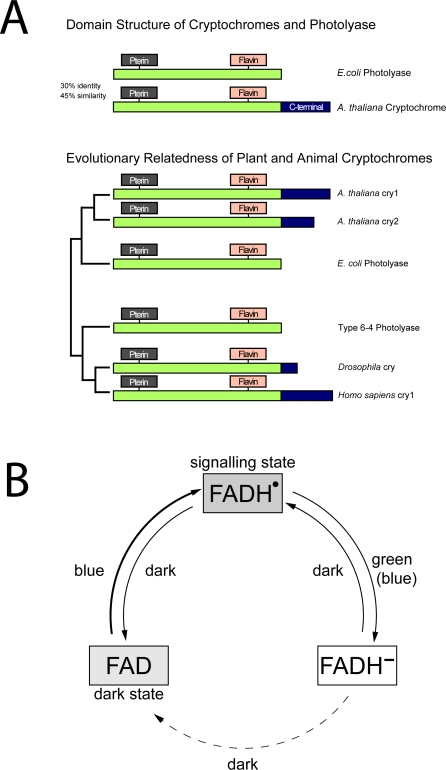
Cryptochromes and DNA Photolyases (A) Evolutionary relatedness and domain structure of plant and animal cryptochromes. N-terminal region is highly conserved and binds the same light-absorbing cofactors: a folate at the N-terminal region and a catalytic flavin chromophore at the C-terminal region. Plant cryptochromes are most related to Type I CPD photolyases, whereas animal cryptochromes are most related to 6–4 photolyases. (B) The photocycle of plant cryptochromes. In the dark, the flavin chromophore is in its oxidized redox state. Blue light induces conversion to a meta-stable semiquinone redox state that is the activated signaling state. Green light causes further reduction to the fully reduced redox state of flavin, which is inactive in signaling. In the dark, fully reduced flavin reoxidizes to the fully oxidized form and can be reactivated by blue light. The photocycle of plant cryptochromes is different from DNA photolyases, in which only the fully reduced redox state is catalytically active.

Subsequent to their discovery in plants, cryptochromes were identified in animal (human and mouse) systems by isolation of homologous cDNAs whose encoded proteins were likewise not functional in DNA repair [8]. Interestingly, these animal-type cryptochromes were more similar to a type of DNA photolyase that repairs 6–4 photoproducts, than to the type I cyclobutane pyrimidine dimer (CPD)-repairing DNA photolyases to which the plant cryptochromes are most closely related. Therefore, animal-type cryptochromes are thought to have evolved independently from different photolyase ancestors than plant cryptochromes [9]. A signaling role for animal-type cryptochromes was first identified in insects, Drosophila melanogaster (D. melanogaster), through isolation of a mutation in the D. melanogaster cryptochrome (Dmcry) resulting in failure to properly entrain the peripheral circadian clock [10,11]. The role of Dmcry as a light-sensing input to the circadian clock is now well established, occurring by interaction of Dmcry with known clock proteins such as timeless or period [12,13]. Thus, although derived from different evolutionary photolyase ancestors, both plant Atcry1 and Dmcry act as signaling molecules that undergo light-sensitive interactions with partners to initiate signaling reactions. A schematic of the basic structural characteristics and evolutionary relation of cryptochromes and photolyases is presented in Figure 1A.

Despite rapid advances in identifying the signaling pathways and molecular targets of cryptochromes in various organisms, the primary light-driven reactions that initiate the signaling process have remained elusive until very recently. Like DNA photolyases, cryptochromes bind flavin adenine dinucleotide (FAD) as a blue-light–absorbing cofactor [1,2]. However, the resting state of flavin in Atcry appears to be the fully oxidized redox form rather than the reduced form as found to be catalytically active in DNA photolyases [14]. Through a combination of in vitro studies with purified proteins and whole-cell in vivo spectroscopic techniques, it was deduced that protein-bound flavin both in Atcry1 and Atcry2 is reduced by blue light through intraprotein electron transfer resulting in accumulation of a relatively long-lived semiquinone intermediate form which is believed to represent the active signaling state [15–18]. This photoreaction is thought to be the basis for conformational changes that occur in the protein to initiate signaling [19,20]. When returned to darkness, plant cryptochromes slowly reoxidize back to the fully oxidized state of the flavin chromophore. In DNA photolyases, a similar light-induced photoreduction, known as photoactivation, can also occur but is relatively unimportant to biological activity since generally the FAD cofactor of DNA photolyase is fully reduced and mostly remains in this redox state independent of light conditions in vivo [21]. Therefore, plant cryptochromes have apparently transformed a minor photoreaction intrinsic to DNA photolyases to form the basis for a novel function as photoreceptor; a summary of the plant cryptochrome photocycle is presented in Figure 1B.

The mechanism of light activation of animal-type cryptochromes is currently unknown. Although structurally similar to plant cryptochromes, the animal proteins apparently stem from 6–4 photolyase-type ancestors and not from CPD photolyases (which are related to plant cryptochromes) [9] and so could have evolved a different photocycle. Nevertheless, purified preparations of Dmcry have been recently shown to undergo a photoreduction reaction similar to plant cryptochromes in vitro, to a relatively stable radical intermediate [22]. Furthermore, studies of wavelength sensitivity show little Dmcry activity above 500 nm (green/red) light [23,24]. These characteristics are consistent with the known mechanism of activation of plant cryptochromes. On the other hand, mutation of conserved tryptophan residues that play a role in photoreduction [21] to redox inactive Phe have not been reported to affect biological activity of either mouse cry [25] or a recently characterized insect cryptochrome from monarch butterfly (Danaus plexippus) (Dpcry1) and from other insects [26,27].

A further complication arose with the identification of a light-independent function for mammalian cryptochromes. Transgenic mice in which both existing cryptochrome alleles (mcry1 and mcry2) had been knocked out showed a complete absence of rhythmic activity. This led to the conclusion that mammalian cryptochrome functions as a central component of the circadian oscillator [28,29]. Like insect cryptochromes, mcry1 and mcry2 were shown to interact with known components of the mammalian clock and thereby obtain a novel biological role. However, these interactions were entirely independent of light [30], and moreover, the clock phenotype of mcry1 and mcry2 occurs in continuous darkness without light interruption over several days. Mammalian cryptochromes are similar to insect cryptochromes and apparently stem from the same 6–4 photolyase ancestor. Moreover, they are flavoproteins and show conservation in amino acids required for light activation in photolyases and other cryptochromes. Despite these similarities, the light-independent nature of mammalian cryptochrome response leads to the question of whether these signaling molecules retain the ability to respond to light at all.

The aim of the present study is to resolve the question of whether and how light activates animal-type cryptochromes. We have employed in vivo spectroscopic techniques including a novel application of electron paramagnetic resonance (EPR) to detect photoconversion of flavin and accumulation of radical in living whole cells. We have examined both Dmcry and Homo sapiens cryptochrome-1 (Hscry1) as representative light-sensitive and light-insensitive cryptochromes, respectively. These experiments showed that light activation of animal cryptochromes occurs by photoreduction and accumulation of a radical signaling intermediate, similar to plant cryptochromes and unlike photolyases. Furthermore, because Hscry1 undergoes the same photoreactions, mammalian cry is demonstrated to have the capacity to function as a light sensor.

## Results

In plant cryptochromes cry1 and cry2, the dark state of the flavin is in the oxidized form in vivo (Figure 1B). To determine the nature of the dark state of flavin in animal-type cryptochromes, we measured a classic action spectrum for Dmcry activity in living flies. An action spectrum is a dose-response curve for photoreceptor sensitivity in which the response of an organism is determined at multiple wavelengths of light and at multiple light intensities at each wavelength. In this way, the response will depend on how well the photoreceptor absorbs light at the given wavelength. The wavelength at which peak activity can be observed in the living organism indicates the absorption maximum (in which the light is absorbed at highest efficiency) of the responsible photoreceptor. If performed to sufficient resolution, such action spectra can be compared to the absorption spectrum of a purified pigment or photoreceptor and in this way identify the nature of the photoactive pigment implicated in a given biological response [6].

**Figure 2:**
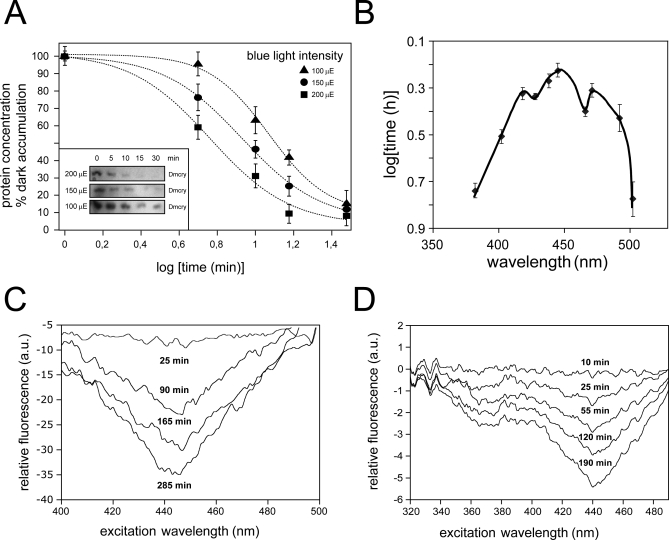
Redox State of Animal Cryptochromes and Photoreduction In Vivo (A) Time course of Dmcry protein degradation in irradiated flies. Irradiation of flies and quantitation of signal was performed as described in Materials and Methods. (B) Action spectrum of Dmcry activity in living flies. *Y*-axis is a measure of activity (irradiance expressed as time to induce 50% Dmcry degradation) at indicated wavelengths. Data was obtained from plots of time courses of Dmcry degradation in Figure S1. (C) In vivo difference spectrum of Dmcry flavin photoconversion. Dmcry-overexpressing insect cells were irradiated with blue light (445 ± 10 nm, 25 μmol m^−2^ s^−1^), and excitation spectra were taken at the indicated intervals by monitoring emission at 525 nm in a fluorescence spectrophotometer. The successive plots were obtained by subtraction of spectra from the (dark) baseline spectrum taken before light treatment (see Figure S2). Peak excitation efficiency at 450 nm decreased as a function of time, consistent with photoreduction of oxidized flavin in vivo. (D) In vivo difference spectrum of Hscry1 flavin photoconversion in vivo. Full-length Hscry1 were irradiated with blue light (445 ± 10 nm, 25 μmol m^−2^ s^−1^), and excitation spectra were taken at the indicated intervals by monitoring emission at 525 nm in a fluorescence spectrophotometer. The successive plots were obtained by subtraction of spectra from the (dark) baseline spectrum taken before light treatment (see Figure S2).

As a possible assay for Dmcry function, we investigated its characteristic property of degradation that followed upon photoreceptor activation. Levels of Dmcry protein decrease rapidly in flies subsequent to blue-light irradiation, likely due to conformational change in the photoreceptor leading to targeting to the proteasome [11,23,31]. This degradation can be quantitatively monitored by western blot analysis with Dmcry antibody. However, in order to be useful for action spectroscopy, the amplitude of the response (decline in Dmcry concentration) must be proportional to the number of photons of light energy absorbed by the photoreceptor, and not simply a delayed response with little direct relation to the light input signal. To test this property, we irradiated living flies for fixed time intervals with blue light (450 nm) and observed decrease in levels of Dmcry protein over time as previously described [11,23,31] (Figure 2A). Importantly, when blue-light irradiation was performed at different blue-light intensities, the time required to reach a given decline in Dmcry protein levels was proportional to the given light intensity. For example, to obtain a decrease to 50% of the original Dmcry protein concentration requires 6.2 min at irradiance of 200 μmol m^−2^ sec^−1^, 9.3 min at 150 μmol m^−2^ sec^−1^, and 12.5 min at 100 μmol m^−2^ sec^−1^ (from Figure 2A), respectively. Therefore, Dmcry degradation obeys the Bunsen-Roscoe law of reciprocity, indicating that it is a response to the total number of photons, independent of irradiance time, and so represents an accurate measure of photoreceptor light responsivity [32].

For generation of this Dmcry action spectrum, living flies were first dark adapted to accumulate maximum levels of dark state cryptochrome. Flies were then subjected to continuous irradiation at a set photon fluence rate of 17 μmol m^−2^ s^−1^ at wavelengths between 380 and 502 nm. Shorter wavelengths are impractical because of the increasing absorption from cell components and therefore increasing errors. Levels of Dmcry protein were monitored by western blot analysis. Illumination time was varied to provide the different irradiance, as permitted by reciprocity. Shorter or longer wavelengths of light proved to be ineffective at eliciting significant response (unpublished data). Dose-response plots of the time course of Dmcry protein degradation at different wavelengths of light showed linear decay as a function of the log irradiation time for points between 20% and 90% of dark levels of protein accumulation (Figure S1). The action spectrum is plotted from these dose-response curves using the total irradiance required to reduce Dmcry protein levels to 50% of dark controls at each wavelength (Figure 2B). The curve is inverted to give a visual image whereby the peak efficiency (the wavelength that required the shortest time to elicit 50% Dmcry degradation) represents the absorption maximum of the responsible photoreceptor. Peak wavelength sensitivity was at 450 nm, with defined shoulders around 420 and 480 nm, matching the spectrum of protein-bound oxidized flavin [33,34]. Oxidized flavin is therefore the likely photoactive pigment of Dmcry in living whole flies, similar to plant cryptochromes [14] and in marked contrast to DNA photolyases in which flavin is fully reduced [2].

We next determined the nature of the chemical reaction induced by light in animal cryptochromes in living cells. We performed baculovirus-driven expression of Dmcry and Hscry1 cryptochromes in Sf21 insect cells, where photoreceptor protein accumulates to sufficiently high levels for direct application of spectroscopic and biophysical techniques in vivo [18]. To verify whether cryptochrome-bound flavin can be directly observed, expressing whole Sf21 cells were harvested and placed intact inside a fluorimeter. Fluorescence emission was measured at 525 nm (characteristic of oxidized flavin) over an excitation range of 400–500 nm. Despite the substantial scatter due to measurements of these living intact cells, there was clearly observable signal increase peaking for excitation at 450 nm in cells overexpressing both Dmcry and Hscry1 as compared to uninfected control cells. These results are consistent with oxidized flavin bound to the dark state of the photoreceptors (Figure S2). These data showing increased oxidized flavin in cryptochrome-expressing cells are in agreement with the resting state of Dmcry determined from action spectroscopy (Figure 2B). Interestingly, mammalian cryptochrome also accumulates in the oxidized form and thereby shows functional similarity to Dmcry and not to DNA photolyases. Similar results have been previously obtained for Atcry1 [18].

To initiate the photochemical reaction, Dmcry- or Hscry1-expressing cells were irradiated with blue light and returned to the fluorimeter at intervals for measurement of excitation spectra. This assay detects change in levels of oxidized flavin in these living cells. For both Dmcry and Hscry1, peak excitation at 450 nm showed a progressive decrease over time that matches the spectra for photoreduction of oxidized flavin (Figure 2C and 2D). This decrease was not due to protein degradation since both Dmcry and Hscry1 protein levels remain stable throughout the time course of illumination in Sf21 cells (Figure S3). Furthermore, flavin reoxidation is observed when illuminated cell cultures are returned to darkness (unpublished data), indicating that no cryptochrome degradation has occurred. Therefore, both tested cryptochromes had undergone a photoreaction in vivo, leading to change in redox state of protein-bound flavin (Figure 2D).

A similar reaction, known as photoactivation, occurs in DNA photolyases, wherein the flavin chromophore is converted to the fully reduced form by an electron transfer reaction ultimately fed by an extrinsic reductant. An intraprotein electron transfer pathway from the protein surface to the buried flavin has been derived for this light-driven reaction in Escherichia coli DNA photolyase (EcPl) based on crystallographic structural information and on a combination of site-directed mutagenesis and spectroscopy [35–38]. This pathway comprises a chain of three tryptophan residues (W382–W359–W306) that are highly conserved throughout the photolyase/cryptochrome family. Recently, a study with purified Atcry1 has demonstrated the functional relevance of this reaction to cryptochrome photoreceptor activity [16] by substitution of redox-inactive phenylalanines for two tryptophan residues, W400 and W324, which are found in the Atcry1 sequence and crystal structure [39] at the homologous positions to W382 and W306 of EcPl, respectively. These mutant proteins (W400F and W324F) lack the predicted electron donor proximal to the flavin (W400) or exposed to the protein surface (W324). Both proteins were found to have impaired electron transfer activity in vitro and reduced biological activity in living plants. To determine whether flavin photoreduction may occur by a similar pathway of intermolecular electron transfer in animal-type cryptochromes, we have made point mutations in Dmcry of two conserved tryptophan residues. One mutation is distal to the flavin in this pathway (W342F), which corresponds to W306 in EcPl and W328F in Dpcry1 [26], respectively. Second, we have introduced a substitution into the middle member of the electron transfer chain of Dmcry, corresponding to W359 of EcPl. The mutant Dmcry proteins were expressed in Sf21 insect cells to high levels and subjected to in vivo fluorescence spectroscopy to follow flavin photoreduction.

**Figure 3:**
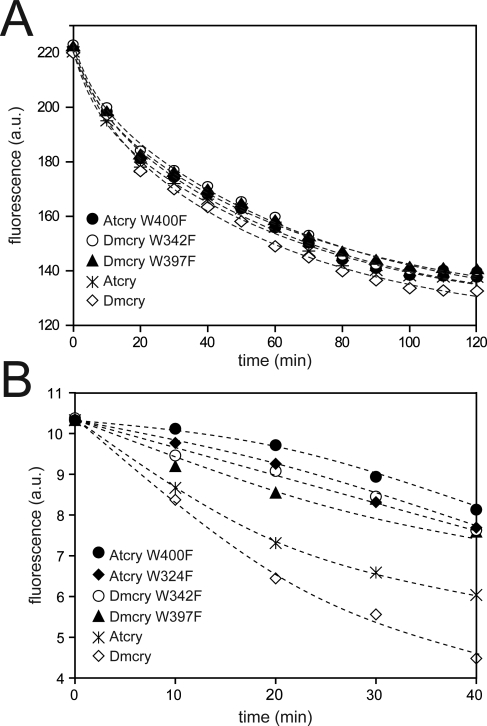
Disruption of the Putative Electron Transfer Chain Alters Photoresponse of Dmcry at Low Photon Fluence Rates (A) Insect cells expressing high levels of either wild-type Dmcry or tryptophan to phenylalanine mutants were irradiated in white light (150 μmol m^−2^ sec^−1^), and excitation spectra taken at the indicated intervals to determine levels of remaining oxidized flavins. Plant cryptochromes (wild-type Atcry1, and W400F and W324F mutants) are included for comparison. (B) Same as in (A) except irradiation was performed at reduced blue-light intensity (450 nm, 10 μmol m^−2^ sec^−1^).

**Figure 4:**
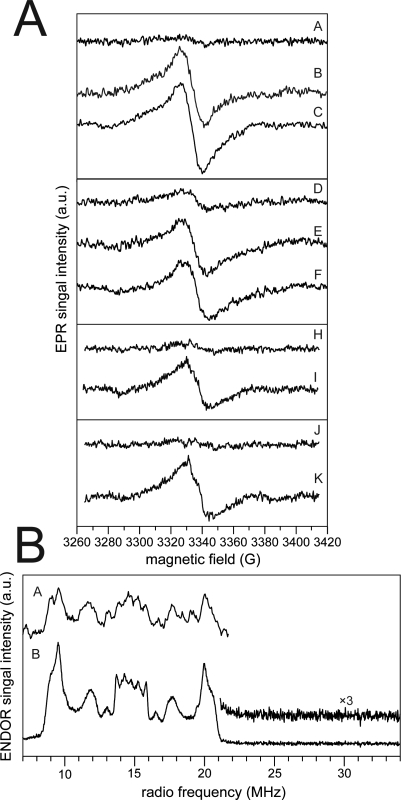
Animal Cryptochrome Photocycle Includes Light-Dependent Radical Accumulation In Vivo (A) X-band continuous-wave (cw)-EPR frozen-solution spectra of intact Sf21 cells expressing Dmcry and Hscry1. A–C, Sf21 insect cells expressing Dmcry after different blue-light illumination times: A, 0 min; B, 3 min; and C, 6 min; D–F, Sf21 insect cells expressing hscry1 after different blue-light illumination times: D, 0 min; E, 6 min; and F, 15 min. Moreover, two Dmcry mutants (W397F and W342F) were recorded under dark and after blue-light illumination conditions: H, 0 min; I, 12 min for W397F; and J, 0 min; K, 12 min for W342F. For more experimental details, see [17,18]. (B) Comparison of X-band–pulsed (Davies) ENDOR spectra for illuminated Dmcry in intact Sf21 insect cells and purified protein. A, cells (sample C in Figure 4A). B, purified protein. The magnified range above 20 MHz (inset trace B) shows the absence of the characteristic ENDOR signal from the N(5)H proton for neutral flavin radicals [54], clearly identifying the radical as anionic flavin species.

For determination of in vivo photoreduction, cryptochrome-expressing Sf21 cells were irradiated with blue light and returned to the fluorimeter periodically to determine remaining levels of oxidized flavins. Unexpectedly, both wild-type and mutant Dmcry proteins showed similar rates of photoreduction in these living cells at high light intensity (150 μmol m^−2^ sec^−1^ white light), as did the W400F mutant of Atcry1 (Figure 3A). This result is surprising since purified preparations of Atcry1 W400F protein and of the W328F Dpcry1 (homolog to Dmcry W342F) showed greatly reduced photoreduction in vitro at even higher light intensities, and there was no significant radical accumulation after this time period [16,26,27]. Therefore, the efficiency of cryptochrome photoconversion in vivo is much higher than that of the purified, isolated protein in vitro, perhaps due to a more conducive redox environment and the presence of relevant electron donors/acceptors in vivo. Nevertheless, at lower photon fluence (10 μmol m^−2^ sec^−1^ blue light), a significant decline in the rate of photoreduction is observed in the phenylalanine mutants of both Dmcry and Atcry1 as compared to wild-type proteins (Figure 3B—see also Figure 4SA and 4SB for experiments performed with further reduced photon fluence). This phenomenon provides a consistent explanation for the observed biological activity of amino acid–substitution mutants in Dpcry and Atcry1. In *Arabidopsis*, biological activity of the mutant proteins (W400F and W324F) was determined at only low blue-light intensity and found impaired at this irradiance in vivo.

To determine the state of the photoreceptor (radical or fully reduced) in the activated cryptochrome, fluorescence emission techniques in whole cells are not sufficient as they can identify only the oxidized form of the flavin chromophore. It cannot, therefore, be concluded from the above studies whether photoreduction in vivo leads to accumulation of a semiquinone intermediate as for plant cryptochromes [16–18], or whether the fully reduced form of flavin accumulates in animal cryptochromes as for DNA photolyases [1,2]. To directly monitor for radical accumulation in response to light in vivo, whole-cell EPR spectra were recorded as previously described [17,18]. Intact Sf21 insect cells with overexpressed Dmcry or Hscry1 protein were irradiated in parallel with nonexpressing control cells at the identical intensities of blue light and rapidly frozen for EPR analysis. A paramagnetic species that does not accumulate in control cells was induced by blue-light irradiation of Dmcry- (Figure 4A, traces B and C) and Hscry1-expressing cells (Figure 4A, traces E and F). This species appears with similar kinetics to both plant cryptochromes [17,18]. Interestingly, there was detectable amount of a radical present even in the dark in some samples (see trace D), although not in all trials, perhaps due to concentrations below our level of detection. This result is in marked contrast to previous experiments, for which radical accumulation was never observed in unilluminated cells [17,18]. Finally, we have examined the *Drosophila* mutant proteins W397F and W342F for radical accumulation in vivo (Figure 4A, traces H–K). Saturating illumination (40 μmol m^−2^ sec^−1^ blue light) leads to accumulation of a radical intermediate form.

To further characterize these signals, X-band–pulsed ENDOR spectroscopy was applied to illuminated whole cells expressing Dmcry (Figure 4B). The observed spectrum in the expressing cells (trace A) is very similar to that obtained from the purified Dmcry protein (trace B). Both spectra differ from those of neutral flavin radicals as seen in plant cryptochromes and corroborate the assignment to an anionic radical species as given previously for the purified protein [22]. Taken together, the in vivo spectroscopic data conclusively indicate that the photocycle for both Dmcry and Hscry1 involves light-dependent flavin reduction and accumulation of the radical state.

Finally, it is necessary to establish the biological relevance of the observed in vivo photoconversion of animal cryptochromes. In plant cryptochromes, the radical state has been demonstrated to be the biologically active signaling state for both Atcry1 and Atcry2 [17,18]. This conclusion resulted from the observation that green light reversed the effect of blue light in the course of cryptochrome activation, due to photoconversion of the active, radical form to the fully reduced, inactive species [17,18,40] (see also Figure 1A). A simple means to determine whether light-induced radical accumulation also has biological relevance for animal cryptochromes in vivo is therefore to measure whether green light (above 525 nm) affects both Dmcry protein accumulation and the kinetics of cryptochrome photoreduction.

**Figure 5:**
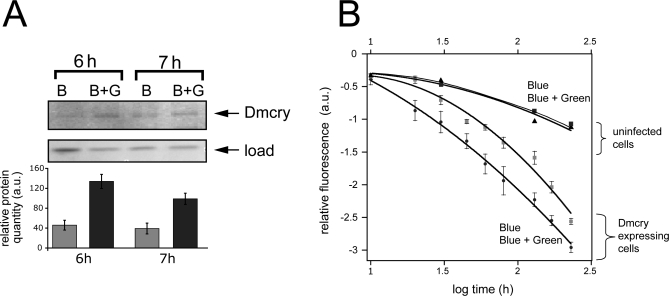
Green Light Alters Cryptochrome Photoconversion In Vivo That Correlates with Function (A) Living flies dark adapted for 3 d were irradiated with either blue (B) (445 ± 10 nm, 10 μmol m^−2^ s^−1^) light or bichromatically with (445 ± 10 nm, 10 μmol m^−2^ s^−1^) plus green (G) (550 ± 25 nm, 50 μmol m^−2^ s^−1^) light (B+G). Western blot with Dmcry antibody was as in Figure 2; equivalent load was verified both by Bradford assay and by staining of total proteins on gels. Three independent trials were performed with similar reduced response to B+G. a.u., arbitrary units. (B) Dmcry photoreduction in living insect cell culture was monitored under conditions of bichromatic irradiation. Peak excitation efficiency at 450 nm was determined in duplicate samples over the indicated time course as above (Figure 1C and 1D). Samples were treated with either B at 25 μmol m^−2^ s^−1^ or bichromatically with B+G (550 ± 25 nm, 150 μmol m^−2^ s^−1^) light over the same interval. Deduced 450-nm peak intensities were plotted over time. No difference was observed in uninfected control cells under conditions of B or B+G irradiation.

To test this prediction, we performed bichromatic irradiation of flies simultaneously with blue and green light (B+G) and compared the response to that obtained with the identical intensity of blue light by itself (B) (Figure 5A). Green-light irradiation by itself resulted in no change in Dmcry protein levels (unpublished data). In each of three independent trials, we observed more rapid decline in Dmcry protein levels in blue light (B) as compared to coirradiation with blue and green light (B+G). This antagonistic effect can only be explained by photoconversion of the (green-light absorbing) radical signaling state to an inactive redox form. In the case of Dmcry-expressing cell cultures, an effect of green light on cryptochrome photoreduction was directly monitored. Cell cultures irradiated with blue and green light (B+G) show accelerated photoreduction of Dmcry as compared to blue light (B) alone (Figure 5B). Although the accumulation of fully reduced flavin can not be directly monitored by this technique, these data are consistent with a shift in the overall flavin photoequilibrium subsequent to formation of the radical, and thereby consistent with the effect of green light on biological activity observed in living flies.

**Figure 6:**
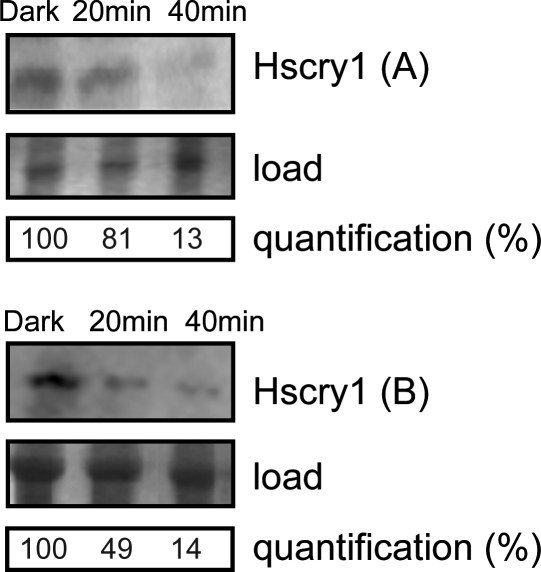
Light Alters Biochemical Properties of Hscry1 in an In Vivo Context Transgenic flies expressing full-length Hscry1 of two independently transformed lines (A: w;31.2A and B: w;26.9A) were assayed for protein expression either in the dark or after the indicated times of irradiation at 150 μmol m^−2^ s^−1^ white light.

Although the present study so far has shown that mammalian cryptochromes undergo similar photoreactions to those of insect and plant, a functional role for light in biological activation remains to be demonstrated. To address this question, we have assayed for a form of activation of Hscry1 in response to light in living flies, where endogenous cryptochrome (Dmcry) is known to undergo light-dependent changes resulting in proteolysis (Figure 2). Transgenic flies expressing full-length Hscry1 under the control of the UAS promoter element were obtained by established procedures (see Materials and Methods). Expression of the recombinant Hscry1 was verified by western blot analysis in two independent transformed lines (A and B). Expressing flies were then dark adapted to accumulate maximal cryptochrome protein and subsequently irradiated with white light. Levels of Hscry1 were assayed during the course of the irradiation. Interestingly, as is the case for Dmcry, significant decrease in Hscry1 protein levels were observed shortly after transfer to white light (Figure 6). These results indicate that Hscry1 undergoes light-dependent proteolysis as does Dmcry in living flies. Since degradation of Dmcry correlates with activation by light and biologically relevant radical formation, a similar mechanism of biological activation is also likely for Hscry1.

## Discussion

In this work, we provide, for the first time, evidence for a photocycle of animal-type cryptochromes such as found in insects and mammals. Cryptochrome-bound flavin is found in an oxidized redox state in vivo, and light activation results in flavin photoreduction to a radical intermediate that represents the likely signaling state. The biological significance of this reaction is supported by the observation of antagonistic effects of green light on Dmcry function, which reduces levels of radical intermediate [17,18,40]. This mechanism contrasts with that of DNA photolyases in which flavin is fully reduced for catalytic activity. Most importantly, Hscry1 from a cryptochrome subfamily with no established light response also has the capacity to undergo this photoreaction in living cells, suggesting the possibility of novel light-sensing capabilities in humans.

A number of studies have indicated that Dmcry responsivity occurs primarily at wavelengths below 500 nm [23,24]. The current study extends these prior observations by providing sufficient fine structure to identify oxidized flavin, with peak of activity at 450 nm and defined shoulders around 420 and 480 nm, as the likely responsible photopigment in the visible range. Further corroboration for the assignment of oxidized flavin as the ground state for animal-type cryptochromes is provided by a classic action spectrum of phase shift in pupal emergence of *Drosophila* [41], a response involving phase shift of the circadian clock which is now known to be under the control of cryptochrome [11]. Consistent with the current work, peak activity was at 450 nm, and the spectrum matches that of protein-bound oxidized flavin.

Interestingly, Dmcry degradation in Schneider cells has been reported to have a peak of activity in the near-UV spectral region [24], whereas in living flies and Dmcry-expressing Sf21 cells, the peak of activity is at 450 nm (see Figure 2B). Like DNA photolyases, cryptochromes are proposed to bind folate derivatives as cofactors in addition to flavin [8]. In DNA photolyases, a folate derivative absorbs light primarily in the near-UV spectral region (370–400 nm) and transfers energy to the flavin chromophore [2]. Recently, a similar role for folate has been postulated in plant cryptochromes [42] whereby light energy for photoreduction is transferred to flavin through a UV antenna pigment. It is therefore likely that the reported near-UV responsivity of Dmcry in Schneider cells also results from light absorption by a folate (or another, yet unspecified) antenna pigment. In that case, absence of near-UV responsivity in Dmcry extracted from whole flies (Figure 2B) suggests that the second chromophore of animal-type cryptochromes may not be available in the majority of insect cell types. This is in line with older experiments done with DNA photolyases, where a low binding constant of the folate chromophore and a therefore heterogeneous folate concentration was concluded.

In plant (Atcry1 and Atcry2) cryptochromes, flavin photoreduction leading to a meta-stable neutral radical accumulation can be observed in in vitro experiments. This property of purified plant cryptochrome contrasts published DNA photolyase data, in which oxidized flavin is rapidly converted to the fully reduced redox state (necessary for DNA repair). Recently, photoreduction experiments were performed with purified preparations of several insect cryptochromes in vitro resulting in similar photoreactions (accumulation of radical and not fully reduced flavin), although the anionic radical, and not the neutral radical, was accumulated [22,26,27]. These results are consistent with the presently derived in vivo photocycle for animal cry activation.

Results from recent studies performed with various insect cryptochromes (fruitfly [Dmcry], butterfly [Dpcry1], mosquito [Agcry1], and silk moth [Apcry1]) [26,27] have called into question the assignment of oxidized flavin as the ground state for animal cryptochromes and argued against a photocycle involving flavin photoreduction. Their interpretation focused on the observation that substitution of amino acids necessary for flavin photoreduction in vitro does not abolish biological activity of these proteins in vivo. This apparent discrepancy between the absence of photoreduction in vitro yet significant biological activity in vivo is resolved by the observation that amino acid substitutions abolishing in vitro photoreduction of purified Dmcry does not, in fact, abolish photoreduction activity in vivo. Photoreduction of oxidized flavin measured by fluorescence techniques (Figure 3A and 3B) in these substitution mutants correlates with concomitant appearance of anionic radical as determined by EPR spectroscopic techniques (Figure 4). The same is true for the W400F mutant of Atcry1, which shows normal rates of photoreduction in vivo under high light intensities even though flavin photoreduction in vitro is virtually zero under these conditions [16]. Cryptochrome photoreduction, therefore, occurs far more efficiently, and by additional alternate pathways, in vivo than is observed for the purified protein in vitro. A similar discrepancy between the light required to activate a purified photoreceptor protein in vitro as compared to activation in vivo has been noted for other photoreceptors, for instance, the class of phototropins [34,43,44] in which blue-light–dependent autophosphorylation requires a much higher irradiance in vitro to obtain a similar effect than is required in vivo.

Results from recent studies showing reduced biological activity at lower light intensity in the W342F mutant of Dmcry [27] are consistent with our observed reduced rates of photoreduction at low photon fluence (Figure 3B). Although quantitation was not formally performed, a prior study in which function of amino acid substitutions in the Trp triad of Dmcry was analyzed [25] also showed apparent reduced biological activity in W-F substitution mutants. In this study, the authors proposed that F can function as electron donor similarly to W, which, however, is not observed [16,26,27]; nevertheless, their data are entirely consistent with the present work. Finally, observations from point mutations that reduce the rate of radical formation in Apcry1 (C402A) abolish protein function in vivo [27] are entirely consistent with our assignment of the radical as the signaling state of the receptor [16]. A proposed mechanism whereby the anionic radical may be the resting state [27] is unlikely given that peak activity is not observed at 470 nm either in the present (Figure 2) or former studies of wavelength sensitivity for this receptor [24,41].

The derived photocycle of animal cryptochromes is therefore similar to the reaction mechanism of plant cryptochromes (Figure 1B). Both photocycles involve reduction of flavin leading to cycling between radical (active) and oxidized (inactive) redox forms. Since these different families of cryptochromes evolved independently from unrelated DNA repair enzyme ancestors, there must be a latent property of DNA photolyases that lends itself to development of photoreceptor properties. The likeliest possibility is that the flavin semiquinone form confers some conformational change on the protein, which can be recognized by yet-unidentified signaling partners and thereby be readily adapted to a role in a signaling pathway. In addition to the classic plant and animal-type cryptochromes, a third family of cryptochromes (cryDASH) has been identified in *Synechocystis* and many other organisms [45]. CryDASH cryptochromes are structurally similar to DNA photolyases, but do not efficiently repair DNA. They evolved from a 6–4 photolyase ancestor but are apparently unrelated to either the plant (Atcry1 and Atcry2) or animal-type cryptochromes described in this study. Although no information as yet exists on the in vivo redox state and photocycle of cryDASH, the purified proteins are converted by light to the fully reduced flavin in vitro [45,46] and retain some single-strand DNA repair activity [47], in this respect appearing more similar to DNA photolyases. It is therefore possible that an additional, entirely unrelated photocycle has evolved for cryDASH-type cryptochromes that is not similar to plant or animal-type cryptochromes.

Perhaps the most intriguing finding of the present study is that mammalian cryptochromes, in particular Hscry1, are responsive to light in vivo. Mammals are generally large, dense (and also often nocturnal) animals, and it makes sense that a molecule such as cryptochrome, which is an essential component of the circadian clock, should be regulated by other means than by direct absorption of light. In fact, with the exception of an isolated report of cryptochrome effect on pupil dilation in mice [48], there has to date been no definite report of light-responsive phenotypes attributed to mammalian cryptochromes at all. Nevertheless, as the present study has shown, Hscry1 can undergo photoconversion in living organisms via a mechanism conserved with that of light-responsive cryptochromes. Hscry1 further undergoes light-dependent proteolysis in living flies, similar to the response mediated by appropriate E3 ubiquitin ligases such as COP1 in the case of *Arabidopsis* cry2 [49], Therefore, the observed light sensitivity of Hscry1 in *Drosophila* is likely to result from surface changes leading to fortuitous recognition of the activated form by a fly E3 ligase. Since Hscry1 is widely distributed in many tissue types of humans, it could be activated by light in the retina or in locations close to the surface, providing the basis for novel biological signaling functions that remain to be determined.

## Materials and Methods

### Interference filters and light sources.

As a light source, white light was produced by slide projectors placed before interference filters of 8–15-nm half-band width (Schott Glaswerke). Filters used in the irradiations for the action spectrum were 380 ± 10 nm, 402 ± 12 nm, 418 ± 13 nm, 428 ± 10 nm, 438 ± 11 nm, 445 ± 10 nm, 466 ± 11 nm, 471 ± 16 nm, 492 ± 15 nm, 502 ± 15 nm, 515 ± 15 nm, and 525 ± 12 nm.

### Irradiation of flies and western blot analysis.

Preparation of fly extracts and detection of dcry was performed essentially as described [50]. Two-week-old adult *Drosophila* (after eclosion) were adapted to dark for 3 d prior to an experiment. Between ten and 15 flies were placed under the indicated wavelengths of light during their subjective night phase (between circadian time [CT] 20–22) and irradiated for the indicated times. Unirradiated control flies did not show variation in Dmcry levels during the time period that illuminations were performed on the test flies. Whole flies were harvested into liquid nitrogen, and extracted proteins prepared as described [50,51]. A total of 20 μg of protein was loaded per lane of an SDS polyacrylamide gel and transferred to PVDF membrane. Protein load was verified prior to load by Bio-Rad Bradford assay and subsequently by Coomassie staining of the blotted gels. Western blot analysis was performed by established methods with affinity-purified anti-Dmcry antibody [51] or Hscry1 antibody to a peptide fragment comprising amino acids 178–194 of the coding sequence (Neosystems). Quantitation of resolved bands was performed digitally using Quantity One imaging software from Bio-Rad.

### Expression of recombinant cryptochrome in insect cell culture.

cDNA for Dmcry and Hscry1 were cloned into a baculovirus transfer vector (pBakPak9; Clontech) by established protocols (Clontech). A histidine tag was introduced upstream of the initial ATG in each construct to allow fast purification. For protein expression, amplified viral supernatant of the recombinant clones were mixed with cell culture and incubated as recommended (Clontech). Protein expression was verified by western blot analysis with appropriate antibody of both whole-cell extract and of proteins purified by metal-affinity chromatography. Presence of flavin was verified by absorption and fluorescence spectra of the purified proteins. For construction of Dmcry mutants W397F and W342F, side-directed mutagenesis was performed by the recommended protocol using Altered Sites II in vitro mutagenesis kit (Promega). The primers designed for mutagenesis are as follows: W397F, GTGCTGCAGTCCATGCTCGAAGCTCTGCCACAA; W342F, CTCGTTCGGCTTAGCGAACGGGATGCTCAGGCA. All clones were sequenced in entirety prior to protein expression.

### Whole-cell fluorescence emission experiments.

Whole-cell fluorescence experiments of Sf21 insect cells expressing recombinant cryptochrome photoreceptors were performed essentially as described [18,42]. Living Sf21 insect cells expressing cryptochrome protein or uninfected controls were centrifuged from culture medium, resuspended in PBS buffer (0.02 M sodium phosphate [pH 7.4], 0.15 M sodium chloride), and placed directly into cuvettes at 10 °C for measurement of fluorescence spectra. Fluorescence emission at 525 nm was monitored in a Varian fluorescence spectrophotometer over a range of excitation wavelengths or at a single designated wavelength as indicated (see Figure 3 legend). Excitation and/or emission spectra were always determined in parallel, both for infected (cryptochrome-expressing) and uninfected cell cultures at identical cell density. For light treatments, samples were removed from the spectrophotometer and placed on ice. Illumination was carried out for the indicated times, using the designated interference filters. Samples were then returned to the fluorescence spectrophotometer to monitor differences in excitation and emission spectra. All experiments were repeated for a minimum of three independent trials.

### EPR spectroscopy.

Sf21 insect cells expressing both Dmcry and Hscry1, control Sf21 cells, and purified Dmcry from Sf21 insect cells were resuspended in phosphate-buffered saline supplemented with 50% (v/v) glycerol in the dark. Aliquots were transferred into EPR quartz tubes (3 mm i.d.) and illuminated for different times at 290 K with blue light (Halolux 100HL; Streppel) using a 420–470-nm band filter (Schott). Samples were then frozen rapidly under illumination in liquid nitrogen and stored therein.

X-band continuous-wave (cw) EPR spectra were recorded using a pulsed EPR spectrometer (Bruker Elexsys E580) with a cw resonator (Bruker ER 4122SHQE) immersed in a helium-gas flow cryostat (Oxford CF-935). X-band–pulsed ENDOR spectra were recorded on the same spectrometer using an ENDOR accessory (Bruker E560-DP), an RF amplifier (Amplifier Research 250A250A), and employing a dielectric-ring ENDOR resonator (Bruker EN4118X-MD-4W1). The temperature was regulated to ±0.1 K by a temperature controller (Oxford ITC-503S). The cw-EPR spectra were recorded at 120 K with a microwave power of 3.0 μW at 9.7 GHz microwave frequency with field modulation amplitude of 0.3 mT (at 100 kHz modulation frequency). For Davies-type ENDOR spectroscopy, a microwave pulse sequence π–T–π/2–τ–π with 64- and 128-ns π/2- and π-pulses, respectively, and a RF pulse of 10-μs duration starting 1 μs after the first microwave pulse were used. The pulse separations T and τ between the microwave pulses were selected to be 13 μs and 500 ns, respectively. To avoid saturation effects due to long relaxation times, the entire pulse pattern was repeated with a low repetition frequency of 200 Hz. Spectra were taken at a magnetic field of 345.7 mT and a microwave frequency of 9.71 GHz.

### Construction of transgenic flies.

The entire coding sequence from the 5′ ATG onwards of *HSCRY1* was introduced behind the upstream UAS promoter element of the pP(UAST) vector via PCR amplification and verified by sequencing; constructs were subsequently introduced into flies by standard methods [51]. Two high-expressing transformed lines with insertions on chromosome II and III, respectively, were selected for further study. Expression from the UAS upstream promoter element was induced by crossing flies to homozygous tim-GAL4 lines expressing the gal4 transcription activator driven by the *TIMELESS* (tim) promoter as described [52]. Parental lines used for crosses were: yw;tim-GAL4 [53] and Hscry1 insertion lines w;31.2A and w;26.9A. Expression was verified in F1 heterozygote progeny by western blot analysis with anti-Hscry1 antibody (monoclonal antibody ref: BIN165979; http://Antikoerper-online.de).

## Supporting Information

Figure S1 Time Course of Dmcry Degradation in Living Flies Recorded at Multiple Wavelengths All irradiations were performed at 17 μmol m^−2^ s^−1^, and differential photon exposure was achieved by varying the time. Plots were prepared from a time course of Dmcry degradation determined by digital quantification of protein signal from western blots. Error bars indicate the standard deviation (s.d.) of three separate determinations. An additional time course at 379 ± 10 nm gave no measurable response in living flies (unpublished data).
(440 KB TIF)

Figure S2 Redox State of Dmcry and Hscry1 Protein In Vivo Living Sf21 insect cells expressing either Dmcry or Hscry1 baculovirus-driven constructs were centrifuged from culture medium, resuspended in PBS (pH 7.4), and placed directly into cuvettes at 10 °C for measurement of fluorescence spectra. (A) Dmcry-infected cells. Fluorescence emission at 525 nm was monitored over a range of excitation wavelengths. Excitation spectra are presented for infected (Dmcry1-expressing) and uninfected cell cultures at identical cell density. Increase in peak intensity at 450 nm is due to oxidized flavin associated with Dmcry in infected cells. (B) same as in (A) but for Hscry1-infected cells.
(299 KB TIF)

Figure S3 Dmcry and Hscry1 Proteins Expressed in Insect Cell Culture Are Not Degraded Subsequent to Light Activation Sf21 insect cells expressing either Hscry1 (upper panel) or Dmcry (lower panel) were harvested either prior to (dark) or subsequent to irradiation for 3 h at 25 μmol m^−2^ s^−1^ of blue light (450 nm). Proteins were resolved and transferred to western blot for probing with affinity-purified Dmcry and Hscry1 antibodies, respectively. Equivalent load was verified by Bradford assay prior to loading samples and by staining of gels (load). Levels of cryptochrome protein remained unchanged subsequent to light treatment.
(320 KB TIF)

Figure S4 Disruption of the Putative Electron Transfer Chain Alters Photoresponse of Dmcry at Lower Photon Fluence Rates (A) Insect cells expressing high levels of either wild-type Dmcry or tryptophan to phenylalanine mutants were irradiated in blue light (450 nm, 5 μmol m^−2^ sec^−1^) and excitation spectra taken at the indicated intervals to determine levels of remaining oxidized flavins. Plant cryptochromes (wild-type Atcry1, and W400F and W324F mutants) are included for comparison.
(B) Same as in (A) except irradiation was performed at higher blue-light intensity (450 nm, 20 μmol m^−2^ sec^−1^).
(844 KB TIF)

## Abbreviations

EcPl: Escherichia coli DNA photolyase
EPR: electron paramagnetic resonance
