# Correction: Human and Drosophila Cryptochromes Are Light Activated by Flavin Photoreduction in Living Cells

**DOI:** 10.1371/journal.pbio.0060200

**Published:** 2008-08-26

**Authors:** Nathalie Hoang, Erik Schleicher, Sylwia Kacprzak, Jean-Pierre Bouly, Marie Picot, William Wu, Alex Berndt, Eva Wolf, Robert Bittl, Margaret Ahmad

Correction for:

Hoang N, Schleicher E, Kacprzak S, Bouly JP, Picot M, et al. (2008) Human and Drosophila cryptochromes are light activated by flavin photoreduction in living cells. PLoS Biol 6(7): e160. doi:10.1371/journal.pbio.0060160

The seventh author's name appears incorrectly. It should be: Alex Berndt.

